# Living with Chronic Heart Failure: Exploring Patient, Informal Caregiver, and Healthcare Professional Perceptions

**DOI:** 10.3390/ijerph17082666

**Published:** 2020-04-13

**Authors:** Natasa Sedlar, Mitja Lainscak, Jerneja Farkas

**Affiliations:** 1 National Institute of Public Health, SI-1000 Ljubljana, Slovenia; natasa.sedlar@nijz.si; 2 General Hospital Murska Sobota, SI-9000 Murska Sobota, Slovenia; mitja.lainscak@guest.arnes.si; 3 Faculty of Medicine, University of Ljubljana, SI-1000 Ljubljana, Slovenia

**Keywords:** heart failure, perspectives, living with chronic disease, qualitative study

## Abstract

Due to the complexity of heart failure (HF) and its treatment process, a high level of patient and informal caregiver engagement is required for management results. We aimed to explore the views of HF patients, informal caregivers, and healthcare professionals about personal experiences, perceived needs, and barriers to optimal HF management. A qualitative study using semi-structured interviews with HF patients (*n* = 32), their informal caregivers (*n* = 21), and healthcare professionals (*n* = 5) was conducted in the outpatient HF clinic in Slovenia in 2018. A content analysis method was used to analyze the data. Negative emotional response to disease and its limitations (especially the inability to continue with work) and changes in family roles were the most prevalent topics regarding the impact of HF on livelihood. Among the most common barriers to HF self-care, were the difficulties in changing lifestyle, financial difficulties due to the disease, traditional cuisine/lack of knowledge regarding a healthy diet and lack of self-confidence regarding physical activity. Despite psychological and social difficulties due to HF being highlighted by patients and informal caregivers, only healthcare professionals emphasized the need to address psychosocial aspects of care in HF management. Established differences could inform the implementation of necessary support mechanisms in HF management.

## 1. Introduction

Heart failure (HF), the only cardiovascular disease with increasing prevalence, is often the final stage of a cardiovascular condition [1,2,3,4]. Due to poor disease outcomes [5,6], impaired quality of life [6,7,8] and commonly occurring multimorbidities of HF patients, complex multidisciplinary treatment, with a high level of patient and informal caregiver participation in the management process is required [3,9,10]. Research [11,12] indicates that active patient participation in care and clinical decision making, based on their disease state and also on their unique cultural background, language, preferences, values, and attitudes, leads to more effective self-management. The studies have, for example, shown that patients diagnosed with HF living in rural areas face particular challenges, such as limited economic resources [13], limited access to management programs [14], lower levels of education and health literacy [15,16] that all lead to lower levels of HF knowledge and worse disease outcomes [17]. Views of patients, informal caregivers and health professionals on challenges involved in the management of HF might however differ, with health professionals often ignoring individual needs, resources and the role of context (such as place of residency, social support, healthcare system) that can affect the capacity for effective HF self-care [18].

To effectively address challenges of the primarily elderly HF population, Coats [19] argues that ‘a more holistic approach to recognizing individual needs and necessary support mechanisms’ (p. L5) is needed in the management of HF, and more studies should assess patient-reported outcomes. In line with the basic principle of patient-centered care, our study took into account the perspectives of patients with HF and informal caregivers. Moreover, we aimed to explore HF patient and informal caregiver experiences of living with HF and their views on treatment and to compare these perspectives with those perceived by healthcare professionals in an outpatient HF clinic in Eastern Slovenia. Understanding individual needs of HF patients and informal caregivers is vital for healthcare professionals and researches because it may lead to more tailored self-care interventions, better health outcomes, and improved HF patient/informal caregiver satisfaction with received healthcare.

## 2. Materials and Methods

### 2.1. Study Design and Participants

A qualitative exploratory research design using semi-structured interviews was used in our study. Participants were recruited from an outpatient HF care setting in a Slovenian general hospital. Eligible patients were adults (over 18 years) with a diagnosis of HF, with New York Heart Association (NYHA) functional class II-III, attending the outpatient HF clinic. Informal caregivers who accompanied patients on their medical visit were asked to participate. Other potentially eligible patients and their informal caregivers were invited to participate by telephone in order to ensure the maximum variation of patients and informal caregivers.

### 2.2. Data Collection

The study was conducted from March to August 2018. The patients, informal caregivers, and healthcare professionals were interviewed using a semi-structured interview guide (Table 1) that was developed by the authors on the basis of the relevant scientific literature (i.e., [20,21,22,23]). It consisted of questions and topics that offered a structure for the discussion during interviews in order to collect similar types of information from each participant. Additional questions were used to clarify and reflect emerging issues raised by participants to further explore their experiences [24,25]. Individual interviews inquired about four main domains of patient and informal caregiver experiences of living with HF: a) main needs, issues and concerns in day-to-day living with HF; b) barriers to HF self-care; c) challenges regarding received psychological and social support; and d) challenges regarding received healthcare in an outpatient HF clinic. Healthcare professionals were invited to talk about their perception of experiences, needs and adopted self-care practices of HF patients. Interviews lasted approximately 15 to 60 min and were conducted at the general hospital by an interviewer with experience in qualitative methods (NS). The number of patients and informal caregivers was determined based on data saturation, i.e., the last five interviews being conducted without additional codes and categories. All interviews were audiotaped and transcribed verbatim.

Demographic information (age, gender, educational level, marital status, employment status, number of years they had been diagnosed with HF, for informal caregivers–relationship to the patient) was collected from the patients and informal caregivers using survey instruments.

### 2.3. Ethical Considerations

All participants gave their written informed consent for participation in the study prior to the study. The study protocol was evaluated and approved by the National Medical Ethics Committee (Approval No. 0120-35/2018/7) and the study was conducted in accordance with the Declaration of Helsinki.

### 2.4. Analysis of Data

Considering the exploratory and descriptive objective of the study, the transcribed interviews were analyzed using a qualitative content analysis method [20,26,27,28]. Each transcribed interview was analyzed as a whole. In the first step, two authors (NS, JF) read the interviews several times to get an overall impression and familiarize themselves with the content. Each of them individually summarized data from the first five interviews: identified individual themes (according to the determined four domains of exploration), openly coded them and suggested groupings of codes under higher-order categories. Once the consensus regarding the list of codes, main categories of the coding scheme and its definitions was reached, one author (NS) coded each interview. Identification of new contents and inconsistencies in the scheme was further discussed to adapt the scheme until all transcripts were coded. Double-coding of some interviews was used to test the inter-rater agreement. Categories and subcategories were examined for patterns and topics, descriptively summarized and compared between all three groups. Additionally, some basic quantification was added by counting categories and subcategories (i.e., the number of participants articulating it) for each group to explore (differences in) predominant and important topics as perceived by different groups.

Descriptive statistics were used to analyze demographic variables. To compare groups of patients with and without an informal caregiver, *χ*^2^ tests to compare categorical variables and Kruskal-Wallis test for continuous, non-normal data were used; *p* < 0.05 was considered statistically significant.

## 3. Results

Overall, 32 HF patients, 21 informal caregivers, and five healthcare professionals were interviewed. The majority of patients were elderly, male, married and retired, while most of the informal caregivers were female, younger than the patients (on average 13 years), married, retired or still employed (Table 2). The majority of HF patients, accompanied during their regular medical visit to the HF clinic (*n* = 18) were male, retired, married or widowed. They were on average almost 10 years older compared to the HF patients that came to their medical visit unaccompanied (*n* = 14). Apart from age and gender, differences in sociodemographic variables between these two groups were non-significant. Informal caregivers, accompanying HF patients to the HF clinic (*n* = 18) were mostly their partners or children that live in the same household. The majority of healthcare professionals were female, three of them were registered nurses and two were cardiologists.

**Figure 1:**
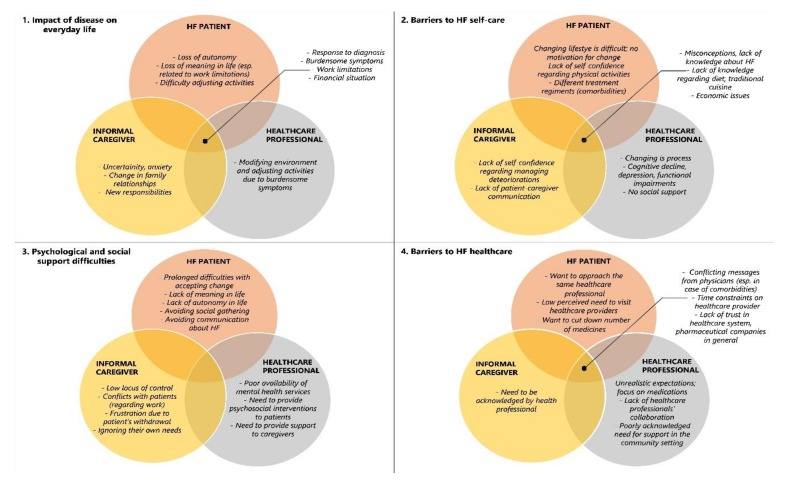
Descriptive summary of similarities and differences in topics from interviews with patients, informal caregivers, and healthcare professionals. Within each domain of exploration, various similar and different topics emerged as relevant to the three interviewed groups.

The main findings, related to four main domains, with illustrative quotations are provided below. For the list of all categories and subcategories generated by qualitative content analysis see Appendix A Table A1. We were particularly interested in whether patients and informal caregivers on one hand and healthcare professionals on the other, agreed regarding experiences, perceived needs, and barriers to optimal HF care. Therefore, Figure 1 descriptively summarizes similarities and differences in topics, emphasized by each group. For the prevalence of categories among all three groups see Appendix A Table A2.

### 3.1. Impact of HF on Everyday Life

Some HF patients (19%) and more than half of the informal caregivers (57%) reported experiencing distressing emotions (i.e., shock, fear, despair, anxiety) after being given the diagnosis (“…*I was shocked at the beginning… when they told me, what has happened*…”). The majority of HF patients (78%) reported that experiencing burdensome symptoms of the disease, such as breathlessness and tiredness, made their life difficult.

A similar proportion (72%) of HF patients emphasized their limited activity, especially limitations or inability to continue with their working activities, as the most difficult aspect of life with HF (“…*everything collapsed, my life turned around, I have to sit still, in front of the TV, on the couch… I used to work for my whole life, from a young age*…”). Loss of independence (34%) and economic difficulties due to their inability to work as before their diagnosis (22%) were perceived as other burdensome aspects of the disease for interviewed HF patients (“…* I can’t work as much as before, I can’t make as much money as I used to–we had to reduce the number of livestock heads *…”), that were mentioned by some (29%) of informal caregivers as well.

On the other hand, the majority (90%) of informal caregivers reported that family roles and relationships have changed after the diagnosis and they had to change their lifestyle in order to adapt to the patient’s limitations (“…*well, of course, it’s hard, our life has changed… we adapted our days according to his /patient’s/ needs … time for lunch, now we go for a walk, we come home… following his schedule… now everything revolves around this*…”). More than two thirds (71%) of the interviewed informal caregivers reported they feel increased responsibility for the patient, i.e., have difficulties leaving the patient alone, monitor the patient all the time, try to be always available.

Burdensome symptoms, work limitations and economic difficulties were mentioned as the most difficult aspects of life with HF by all interviewed healthcare professionals. All of them also mentioned that patients modify their environment and daily activities to adapt to burdensome symptoms.

### 3.2. Barriers to HF Self-Care

Lack of knowledge and misconceptions about the disease were noticed in some patients’ (16%) and caregivers’ (19%) answers (“…This is not a serious disease; she doesn’t have urinary incontinence, uses the bathroom independently, eats normally, brings food, has no problem with other basic daily activities…”) and were recognized as an important barrier by healthcare professionals as well.

On the other hand, more than half (60%) of the patients emphasized they have knowledge about the disease needed for self-care but have to some extent difficulties adhering to the recommended self-care behaviors (“…*everything sounds nice, everything is easy to understand… but it is difficult to do *…”). Difficulties of changing life habits were also acknowledged by healthcare professionals, especially registered nurses, that offer educational and psychosocial support to patients (“…*I think, we all find it difficult to change our habits, this is a process that takes time*…”).

Most of the patients (81%) reported compliance with the medical regimen, the majority (59%) to self-monitoring weight and pulse. Almost a third (31%) of patients and some (19%) informal caregivers reported that eating a healthy diet and avoiding salt was more difficult for them due to insufficient knowledge (i.e., how much salt is contained in processed food) as well as traditional cuisine (don’t want to prepare food that is not salty/tasty, it is difficult to avoid pork meat) or practical reasons (cannot afford to cook separate meals to the patient). These aspects were acknowledged by all interviewed healthcare professionals. Regarding physical activity, almost half (44%) of interviewed HF patients reported moderate physical activity while others reported primarily resting and relaxing, avoiding exercise in the winter to avoid flu, not being self-confident regarding how much effort is allowed. Informal caregivers were mostly aware of recommendations regarding regular physical activity but 24% of them reported they do not want to push patients too much. Only some healthcare professionals addressed these issues.

Almost half of the interviewed patients (44%) stated that comorbidities are complicating their attempts to manage HF (“…I drink 1.5 L of water per day, I should not drink more… but I should drink more water because of my kidney disease… now I don’t know what to do…”). This aspect was emphasized by healthcare professionals as well; depression, cognitive decline, and chronic kidney disease were among the most commonly mentioned comorbidities.

Healthcare professionals recognized the financial and economic issues (low retirement income/social support, lower-income due to inability to work /losing job) affecting the patient’s self-care, i.e., cost of healthier food choices, cannot grow their own food anymore, cost of transportation to medical appointments, do not have enough income to buy scales/blood pressure monitor (“…*I still see that a substantial number of patients have economic problems. They can’t pay for medical equipment (scales, blood pressure monitor). They always have to ask someone to drive them to medical appointments. Sometimes I get the feeling, they try to hide something from us and do not tell us everything*…”).

The majority (95%) of informal caregivers stated that they offer practical (i.e., make sure patient takes his medicines, prepare meals) support to patients, some of them (33%) also mentioned they offer emotional support (i.e., always check on the patient, ask him how he feels), but healthcare professionals noticed that some patients have no one to care for them.

### 3.3. Psychological and Social Support Difficulties

More than two thirds (69%) of patients with HF reported experiencing negative emotions (helplessness, despair, frustration, anxiety, feelings of uselessness) due to physical and work restrictions. Four of them even reported they developed depression or explicitly mentioned experiencing depressive symptoms as a consequence of the disease. Lack of autonomy and feelings of dependency that made them feel frustrated and guilty for being a burden to others (“…* I can no longer work. So my relatives have to work instead of me… my son became a victim* …”) were mentioned by almost a third (31%) of patients. Three patients reported they socially isolated themselves from others as a consequence of HF, not even sharing experiences with their closest relatives or friends (“… *I no longer like visiting others, they said I’ve isolated myself*…”). One patient even mentioned that being misunderstood by others, i.e., neighbors, people from the village (who do not know the severity of HF) was the most difficult aspect of the disease for him to deal with (“…*I can’t work. My condition is not something that can be seen. And others think I don’t want to work, that’s the worst* …”).

Almost half (48%) of informal caregivers reported experiencing anxiety, especially about the future and their ability to manage sudden deterioration (“… *that’s what I’m afraid of, when it comes to something. That’s what scares me. And it is in my mind every day, you know*…”). Almost two thirds (62%) of informal caregivers indicated that caring for the patient was sometimes frustrating, especially when they felt that the patient does not want to discuss HF and/or his daily condition, withdraws, does not want to be controlled or does not want to stop working (even when feeling worse). Arguments regarding work were mentioned by a third (33%) of informal caregivers. In some cases (19%), arguments that were caused by the patient’s perception of the caregiver being too controlling were mentioned as well (“…*when he goes upstairs to his office, and there aren’t any sounds of walking, coughing etc. I sometimes call him and he tells me to leave him alone…if I need to control his every move*…”). Almost a third (29%) of informal caregivers stated that their needs are less or not important compared to the needs of the HF patient (“…*I am not important now. Often my colleagues and neighbors say to me, that I should look after myself as well*…”). They usually discuss the disease with the patient or someone from the closer family, but do not feel the need to discuss their situation with neighbors and the broader social circle.

**Figure 2:**
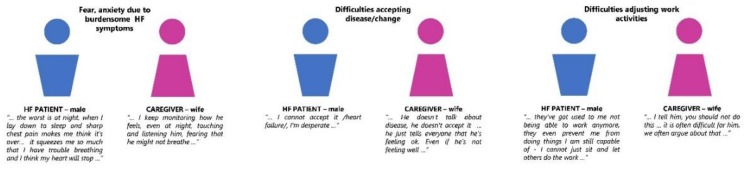
Various effects of heart failure on dyadic interactions and relationships emphasized by interviewed patients and informal caregivers (especially in patient-spouse dyads), that rarely emerged in interviews with healthcare professionals.

Healthcare professionals briefly mentioned some aspects of psychological and social support difficulties that were thoroughly described by HF patients and informal caregivers. Figure 2 shows the effects of these experiences on their dyadic relationships, which were commonly reported by patients and informal caregivers, but not by healthcare professionals.

Healthcare professionals, on the other hand, stressed that access to mental health services is insufficient and psychosocial support for patients and informal caregivers should be available; these aspects were not mentioned by interviewed patients and informal caregivers.

### 3.4. Barriers to Effective Healthcare in HF Clinic

Patients were generally satisfied with received healthcare in the outpatient HF clinic; more than half (60%) of them expressed they received sufficient information and/or practical self-care skills. Moreover, most of them found healthcare professionals easy to approach. A few of them expressed their satisfaction with being able to approach the same healthcare professional over time. On the downside, some patients stressed the negative impact of conflicting messages received from different physicians (especially in the case of comorbidities), mentioned time constraints on healthcare professionals (“…*You know how things are when you go to the doctor–you wait and wait … there is no other option because so many of us are sick*…”), their efforts to avoid visiting doctors, and their concerns regarding pharmacotherapy (i.e., taking too many pills, side effects). Three patients also stressed that no doctor can help them with their condition (“…*No doctor can fix your heart, as you can get your problem with your car fixed in the garage. This is where your condition slowly gets worse and when it comes to a certain point*…”).

Some informal caregivers (19%) expressed their need to be acknowledged by healthcare professionals. However, more than two-thirds of them (72%) mentioned they feel appropriately addressed and are satisfied with the received education about the disease and nursing support.

Healthcare professionals described that patients sometimes had unrealistic expectations about diagnosis/treatment (“…of course, some also expect from the healthcare system to get one pill and everything will be fine. Which is also not realistic…”). They stated that patients asked them mainly about pharmacotherapy, mostly expressing concern over the number of medicines they have to take and their side effects. They noticed the lack of trust in the healthcare system and pharmaceutical companies in general and emphasized insufficient collaboration between healthcare professionals. They were united in agreeing that there is a need for support of HF patients in the community setting (i.e., support in housekeeping, availability of cardiac exercise groups etc.) that has not been properly addressed so far.

## 4. Discussion

In this qualitative study, we explored the perspectives of HF patients, informal caregivers and healthcare professionals on their experiences, perceived needs and barriers to optimal HF management. Among all three groups, lack of knowledge about HF, economic issues, traditional cuisine/lack of knowledge regarding diet, and difficulties in changing lifestyles were acknowledged as important barriers to HF self-care, corroborating previous research findings regarding the complexity of the self-care process [20]. Regarding barriers to effective HF healthcare, all three groups identified the management of comorbidities, conflicting messages from physicians, time constraints on healthcare providers, and a lack of trust in the healthcare system in general. Poor communication [29] that has been suggested to impair disease management [30,31], was identified as an important barrier to HF management by some patients and informal caregivers–mostly with regard to the patient-caregiver relationship (i.e., interpersonal conflicts due to work limitations, inconsistencies in views about managing illness), only in rare cases with regard to the healthcare system or providers. Aspects that were particularly salient among healthcare professionals were unrealistic patient expectations and the need to systematically address the psychosocial needs of patients and informal caregivers in the healthcare system and the community setting.

In agreement with previous studies [7], our results show that interviewed HF patients and their informal caregivers reported various distressing emotions (frustration, anxiety, depressive symptoms) after being diagnosed with HF. Burdensome symptoms and the inability to continue with work as before their diagnosis made accepting the disease especially difficult for interviewed HF patients and often increased strain on their finances [32]. Overall, the importance of continuing with work (continuing with their job, working on the land, housework) seemed to be a prevalent topic emphasized by the majority of patients with HF in this geographic region [33], where work is considered an integral part of life and one of the most important values. This aspect was recognized by healthcare professionals as well. What is more, some patients reported they socially isolated themselves because of HF (i.e., avoiding social activities, avoiding discussing their HF with relatives, friends, neighbors) or felt misunderstood by others (i.e., fear of being perceived as lazy).

Patients with HF and their informal caregivers often mention their life patterns and roles were changed due to the disease. In line with findings from Yeon and colleagues [34], patients in our study emphasized feelings of uselessness, helplessness, sometimes isolation; the majority of them expressed frustration regarding their inability to work and loss of autonomy, arguments with informal caregiver were mentioned as well. Similar to other studies (i.e., [35]) informal caregivers in our study reported experiencing increased responsibility for the patient, neglecting their needs, monitoring the patient all the time. Feelings of uncertainty were commonly mentioned as burdensome, along with feelings of frustration by the patient’s withdrawal, unwillingness to discuss their condition or sometimes stubbornness (i.e., continuing with work even when experiencing worsening symptoms). This is especially relevant because higher caregiver strain has shown to be associated with worse outcomes of patients and informal caregivers [36]. Findings from interviews with healthcare professionals regarding difficulties of dealing with burdensome symptoms, physical and work limitations due to HF were similar to those identified by patients and informal caregivers. Therefore, it comes as no surprise that they recognized psychosocial support as an important aspect of HF management, enhancing the health-related quality of life of patients and informal caregivers. Similar findings regarding the importance of addressing the social and emotional aspects of adjustment to HF as a part of self-care interventions were emphasized by other authors (i.e., [37]). Surprisingly, the need for psychosocial support was not mentioned as an important or necessary part of HF management by the majority of patients and informal caregivers. This could be due to the perception that traditional medicine and the healthcare system are dealing with physical health problems only.

In contrast to the interviewed patients and informal caregivers, the healthcare professionals thought that patients sometimes have unrealistic expectations about treatment, relying on pharmacological treatment too much, modifying environment and activities to cope with burdensome symptoms, while not being motivated (enough) for lifestyle changes. The topic of motivation was identified by patients as well; more than half of them stated they received the needed HF self-care knowledge but find it difficult to adhere to the recommendations [33]. Other factors that hindered them from adhering to self-care recommendations were similar to those identified in other studies (i.e., [38]), including comorbidities, depression, lack of social support and economic issues. The latter was a commonly mentioned barrier to HF self-care [39], especially by healthcare professionals (i.e., the patient cannot afford to pay for healthy food, transportation, medical equipment), which is not surprising, given the socio-economic standard in this part of Slovenia is one of the lowest [40]. Among contextual factors that were emphasized among HF patients in this geographic region were preferences for traditional foods that made it difficult to adhere to a healthy diet [41]. The importance to continue with work as before the disease, causing them distressing emotions and making it difficult to accept and adjust to the disease, was another commonly mentioned contextual factor [33]. Another important barrier to effective HF management identified by some informal caregivers was the lack of communication about HF in the dyad, mostly due to the patient’s unwillingness to discuss the condition. Similarly, healthcare professionals noticed that patients are often not willing to share their personal struggles with them, making their interpersonal sensitivity and good communication skills even more important aspects of healthcare [42].

Our results show that interviewed HF patients and informal caregivers were mostly satisfied with received healthcare. In agreement with previous studies (i.e., [43]), all three groups emphasized factors related to the healthcare system (i.e., time constraints on healthcare professionals, uncoordinated care by healthcare professionals in different specialties), but also mentioned individual factors (i.e., lack of trust in the healthcare system). Results from our study also indicate caregiver satisfaction with received education about the disease and nursing support. However, most of them reported neglecting their needs, so it might be possible that some of them are not even aware of their actual needs and possibilities for support in the healthcare or community setting. We believe that offering a supportive environment and facilitating discussion and sharing problems in education for HF patients and informal caregivers could further support their ability to deal with HF and enhance their quality of life [44]. Healthcare professionals also recognized patients’ and informal caregivers’ need for psychosocial support and emphasized that efforts to offer them support in the community setting should be made in the near future. However, as these aspects of adjustment to the disease were not recognized as important by patients and informal caregivers, raising awareness and gradual implementation of interventions is needed.

### Strengths and Limitations

The study was conducted by an interprofessional team of researchers with different backgrounds, allowing for a multi-disciplinary integration of perspectives and opinions. Our sample size could appear as insufficient, but when taking a closer look at the other published qualitative studies concerning the experience of living with HF (i.e., see Yeon and colleagues for the review [7]), only around one-third of studies reported sample sizes above 20 participants. Additionally, consensus regarding data saturation was made by our research team. However, the number of healthcare professionals was smaller due to practical reasons, i.e., trying to include all three groups from one HF clinic to be able to directly compare their perspectives.

Our sample includes patients from the socioeconomically disadvantaged area of Eastern Slovenia, therefore, the patients in our study may not comprise a representative sample of residents with HF in Slovenia. Additionally, findings obtained from qualitative research may not be generalizable in the same way as quantitative and should be interpreted with caution.

Future research should focus on developing interventions that could address the barriers experienced by patients, informal caregivers, and healthcare professionals. It might also benefit from a longitudinal design to explore changes in HF management barriers perceived by patients and informal caregivers [45].

## 5. Conclusions

Our study contributes to an understanding of the ways patients, informal caregivers, and healthcare professionals differ in their perspectives on living with HF and the perceived needs. The majority of topics emphasized as important by HF patients and informal caregivers were also recognized by healthcare professionals. However, only healthcare professionals recognized the importance of addressing the psychosocial aspects of care in HF management. Established differences should be taken into account when planning future service configuration and intervention strategies, especially in environments with adverse socioeconomic circumstances.

## Figures and Tables

**Table 1:** Patient and informal caregiver interview domains and sample questions (adapted from [20,21,22,23]).

Interview Domain	Sample Question
General questions about the disease	–Tell me about heart failure and how you/patient found out you had it?
	–How does your present life differ from your life before heart failure?
General questions about self-care	–What are you doing on a daily basis to manage your condition/help patient (dealing with symptoms, specific tasks–taking medication, monitoring weight, managing appointments etc.)?
	–What makes it easier/harder to take care of yourself/patient?
	–How do you know you’re/patient’s doing ok, what do you do to monitor yourself/patient?
General questions about psychological, social support	–How does heart failure affect the relationship with your relatives/friends/family?
	–Who do you talk to about (issues mentioned throughout the interview)?
	–What would you do if you/patient felt down, frustrated or worried?
General questions about received healthcare in an outpatient heart failure clinic	–How do you experience your encounters with healthcare?
	–Which information have you received by healthcare professionals?
	–What instructions did you receive about taking care of yourself/patient at home? Do you think the instructions are easy to understand, what would help you fit the instructions into your normal activities better?
	–How comfortable are you talking to your physician about your/patient’s health concerns?

**Table 2:** Participant characteristics.

Demographics	Patients *n* = 32	Informal Caregivers *n* = 21	Patients with Informal Caregiver *n* = 18	Patients without Informal Caregiver *n* = 14	*p*-Value (Patients with-without Informal Caregiver)
**Gender**, *n* (%)					
Male	20 (63)	4 (19)	14 (77)	6 (43)	*p* = 0.018
**Age**, M ± SD	72.9 ± 11.3	59.7 ± 13.7	77.1 ± 7.6	67.7 ± 13.3	*p* = 0.013
**Educational Level**, *n* (%)					
Incomplete primary	2 (6)	1 (5)	1 (6)	1 (7)	*p* = 0.081
Primary school	14 (44)	5 (24)	9 (50)	5 (36)	
Vocational	9 (28)	4 (19)	5 (28)	3 (21)	
Secondary	2 (6)	8 (38)	1 (6)	1 (7)	
College	1 (3)	0	0	1 (7)	
University	1 (3)	2 (10)	1 (6)	0	
**Marital Status**, *n* (%)					
Single/never married	5 (16)	3 (14)	2 (11)	3 (21)	*p* = 0.831
Married	19 (59)	16 (76)	11 (61)	8 (57)	
Divorced/separated	1 (3)	1 (5)	2 (11)	0	
Widowed	5 (16)	0	3 (17)	1 (7)	
**Employment Status**, *n* (%)					
Full-time	1 (3)	6 (29)	1 (6)	0	*p* = 0.210
Part-time	0	1 (5)	0	0	
Retired	25 (78)	9 (43)	16 (89)	9 (64)	
Unemployed	2 (6)	2 (10)	0	2 (14)	
Others	1 (3)	3 (14)	0	1 (7)	
**Years Since Heart Failure Diagnosis**, M ± SD	5.8 ± 5.1		6.5 ± 5.8	4.7 ± 4.1	*p* = 0.282
**Relationship to Patient**, *n* (%)					
Husband/Wife/Partner		10 (48)	9 (50)		
Son/Daughter		8 (38)	7 (39)		
Other relative^1^		2 (10)	1 (6)		
Friend		1 (5)	1 (6)		
**Lives in Same Household**, *n* (%)					
Yes		15 (71)	14 (78)		
No		5 (24)	4 (22)		

^1^ Grandchild, daughter-or son-in-law. Note. Percentages may not total 100 due to missing data or rounding.

## Appendix A

**Table A1:** Categories and subcategories generated by qualitative content analysis.

Categories	Subcategories
1. Impact of disease on everyday life	
Immediate response to diagnosis	–Shock
	–Denial
	–Anger
	–Sadness
	–Fear, anxiety
Burdensome symptoms	–Fatigue
	–Shortness of breath
	–Swelling
	–Weight gain
	–Lack of appetite
Limitations	-Physical
	–Work-inability to continue with job
	–Work-inability to continue with domestic work, farming
Quality of life	–Loss of autonomy
	–Loss of meaning
	–Uncertainty, anxiety
	–Social isolation
Change in lifestyle	–Modifying environment
	–Adjusting activities
	–Change in family relationships
	–Change in work-family balance
	–New responsibilities
Financial situation	–Lower income
	–More expenses
2. Barriers to self-care	
Self-care skills	–Medication taking–low
	–Self-monitoring–low
	–Adherence to recommendations - low
Knowledge	–Misconceptions about heart failure
	–Lack of knowledge–heart failure in general
	–Lack of knowledge–self-care recommendations
Motivation	–No motivation
	–Change is difficult
	–Change is a process
Self-confidence	–Physical activity
	–Managing deterioration
Comorbidities	–Different treatment regiments
	–Functional impairments
	–Cognitive decline
	–Depression
Social support	–No social support
	–Lack of patient-caregiver communication
Social context	–Traditional cuisine
	–Social pressure
Economic issues	–Extra cost–food
	–Extra cost–devices–Extra cost–transportation
3. Psychological, social support difficulties	
Psychological difficulties	–Prolonged experience of psychological distress
	–Prolonged difficulties with accepting change
	–Lack of meaning in life
	–Lack of autonomy
	–Low locus of control
	–Ignoring own needs
Social/relationship difficulties	–Avoiding social gatherings
	–Avoiding communication about heart failure
	–Conflicts
	–Lack of emotional support
Need for organized psychosocial interventions	–Mental health services to chronic patients in hospitals
	–Psychosocial interventions for heart failure patients
	–Psychosocial interventions for informal caregivers
4. Received healthcare in outpatient heart failure clinic	
Satisfaction with received healthcare	–Obtained knowledge–low
	–Obtained self-care skills–low
Organizational issues	–Conflicting messages from different specialists
	–Lack of collaboration between different healthcare professionals
	–Time constraints
	–Not being able to approach the same healthcare professional
Attitudes towards healthcare	–Lack of trust–healthcare
	–Lack of trust–pharmaceutical companies
	–Low perceived need to visit healthcare professionals
	–Unrealistic expectations
Need for supportive environment in heart failure management	–Lack of (interpersonal) skills by healthcare professionals
	–Lack of resources by healthcare professionals
	–Need for inclusion of informal caregivers in healthcare
	–Need for support in the community setting

**Table A2:** Quantification of categories.

Categories	Frequencies *		
	Patients	Informal Caregivers	Healthcare Professionals
1. Impact of disease on everyday life			
Immediate response to diagnosis	[6/32]	[12/21]	[3/5]
Burdensome symptoms	[25/32]	[5/21]	[5/5]
Limitations	[23/32]	[5/21]	[5/5]
Quality of life	[11/32]	[15/21]	[3/5]
Change in lifestyle	[23/32]	[20/21]	[5/5]
Financial situation	[7/32]	[6/21]	[5/5]
2. Barriers to self-care			
Self-care skills	[6/32]	[9/21]	[5/5]
Knowledge	[5/32]	[4/21]	[4/5]
Motivation	[19/32]	[6/21]	[5/5]
Self-confidence	[6/32]	[6/21]	[2/5]
Comorbidities	[14/32]	[4/21]	[5/5]
Social support	[2/32]	[6/21]	[2/5]
Social context	[12/32]	[7/21]	[5/5]
Economic issues	[8/32]	[4/21]	[5/5]
3. Psychological, social support difficulties			
Psychological difficulties	[22/32]	[11/21]	[3/5]
Social/relationship difficulties	[11/32]	[13/21]	[3/5]
Need for organized psychosocial interventions	[0/32]	[0/21]	[5/5]
4. Received healthcare in outpatient heart failure clinic			
Satisfaction with received healthcare	[13/32]	[6/21]	[2/5]
Organizational issues	[5/32]	[2/21]	[5/5]
Attitudes towards healthcare	[7/32]	[3/21]	[4/5]
Need for supportive environment in heart failure management	[5/32]	[4/21]	[5/5]
